# Spillover effects from a type 2 diabetes integrated model of care in 22,706 Australians: an open cohort stepped wedge trial

**DOI:** 10.1186/s12902-024-01692-4

**Published:** 2024-09-10

**Authors:** Shamasunder Acharya, Rachael Taylor, Martha Parsons, John Attia, Lucy Leigh, Christopher Oldmeadow, Katie Wynne, Christopher Rowe, Morag Joseph, Judy Luu, Annalise Philcox, Damien Jackel, Tuan Quach, Christy Sankoorikal, Simone Dagg, Alexis Hure

**Affiliations:** 1 Hunter New England Health District, John Hunter Hospital, Lookout Road, New Lambton Heights, NSW 2305 Australia; 2 School of Medicine and Public Health, University of Newcastle, University Drive, Callaghan, NSW 2308 Australia; 3 Hunter Medical Research Institute, New Lambton Heights, NSW 2305 Australia; 4 Clinical Research Design IT and Statistical Support (CReDITSS) Unit, Hunter Medical Research Institute, New Lambton Heights, NSW Australia; 5 Hunter New England Central Coast Primary Health Network, PO Box 2288, Dangar, NSW 2309 Australia

**Keywords:** Clinical trial, Diabetes mellitus, Type 2, General practice, Health services, Outcome assessment

## Abstract

**Background:**

Many Australian adults are not receiving timely or effective diabetes management to prevent or delay the onset of diabetes related complications. Integrated care, a worldwide trend in healthcare reform, aims to reduce the fragmented delivery of health services and improve outcomes. This study aimed to test whether a specialist-led integrated model of care provided to a small subset of patients in general practices leads to spillover clinical improvements in all patients of the practice with type 2 diabetes.

**Methods:**

Seventy-two general practice sites (clusters) in New South Wales, Australia received the Diabetes Alliance intervention, creating a non-randomised open cohort stepped wedge trial. The intervention comprised of case conferencing, delivered directly to a small proportion of adults with type 2 diabetes (*n* = 1,072) of the general practice sites; as well as practice feedback, education and training. Spillover clinical improvements were assessed on all adults with type 2 diabetes within the general practice sites (*n* = 22,706), using practice level data recorded in the MedicineInsight electronic database, compared before and after the intervention. Outcome measures included frequency of diabetes screening tests in line with the Annual Cycle of Care, and clinical results for weight, blood pressure, HbA1c, lipids, and kidney function.

**Results:**

Compared to before Diabetes Alliance, the odds of all practice patients receiving screening tests at or above the recommended intervals were significantly higher for all recommended tests after Diabetes Alliance (odds ratio range 1.41–4.45, *p* < 0.0001). Significant improvements in clinical outcomes were observed for weight (absolute mean difference: -1.38 kg), blood pressure (systolic − 1.12 mmHg, diastolic − 1.18 mmHg), HbA1c (-0.03% at the mean), total cholesterol (-0.11 mmol/L), and triglycerides (-0.02 mmol/L) (*p* < 0.05). There were small but significant declines in kidney function.

**Conclusions:**

Integrated care delivered to a small subset of patients with type 2 diabetes across a large geographic region has spillover benefits that improve the process measures and clinical outcomes for all practice patients with type 2 diabetes.

**Trial registration:**

ACTRN12622001438741; 10th November 2022, retrospectively registered: https://www.anzctr.org.au/ACTRN12622001438741.aspx.

**Supplementary Information:**

The online version contains supplementary material available at 10.1186/s12902-024-01692-4.

## Background

Half a billion people are living with diabetes mellitus worldwide, with projections of a 50% increase by 2045 [1]. Type 2 diabetes constitutes approximately 90% of all cases of diabetes [1]. In Australia, type 2 diabetes is the 12th largest contributor to the burden of disease, affecting 5.3% of adults [2, 3]. However, 65% of cardiovascular deaths in Australia occur in people with diabetes or prediabetes [4]. The prevalence of type 2 diabetes is also substantially underdiagnosed [5], masking the true burden of disease [6].

For over a decade the International Diabetes Federation has recommended an annual review of type 2 diabetes control and complications, an agreed and updated diabetes care plan, and involvement of a multidisciplinary team in delivering that plan, centred around the person [7]. In Australia, the *Annual Cycle of Care* provides minimum standards for diabetes care, including routine tests and measurements (usually at six or 12 monthly intervals) to identify problems early with the intention of reducing diabetes-related complications [8]. Interventions that improve glycaemia, blood pressure, and lipids have been shown to reduce the risk of premature mortality and cardiovascular disease [9]. The Annual Cycle of Care is intended to be delivered by general practitioners (GP) and other diabetes specialists, based on recommendations outlined in the *Management of type 2 diabetes: A handbook for general practice* [10]. Sainsbury et al. (2018) have reviewed existing evidence to determine what proportion of people with diabetes in Australia receive the Annual Cycle of Care [9]. From the four identified studies, completion rates ranged from 0.9% in an Indigenous population to 37% over a 12 to 18-month period in non-Indigenous people [9]. Sainsbury et al. (2018) also identified 31 studies investigating the proportion of people meeting the HbA1c target of ≤ 7.0%, finding an overall mean of 53% (range 13–79%) [9]. These findings highlight the need for services that focus on better implementation of the minimum standards of care for diabetes, with evaluation of the clinical outcomes [11].

The Diabetes Alliance commenced in 2015, delivering an integrated model of care that provides access to tertiary specialist services within the general practice (or primary care) setting [12]. The Diabetes Alliance was initially piloted across 20 general practices within Hunter New England Local Health District (HNELHD), Australia [12] and has since been implemented on a larger scale involving 120 practices. While Diabetes Alliance has been shown to be effective in enhancing adherence to the Annual Cycle of Care and improving glycaemic control for those who receive the specialist-led case conferencing [12], the question remained whether the learning and knowledge from Diabetes Alliance was transferred and applied by the GPs to their other patients with diabetes, in epidemiological terms a spillover effect [13]. Therefore, this study aimed to evaluate the spillover effect of the Diabetes Alliance on diabetes care for patients with type 2 diabetes seen in the general practice setting.

## Methods

### Study design

This is a non-randomised open cohort stepped-wedge trial comparing clinical and process outcomes before and after Diabetes Alliance (also referred to as the intervention). The trial was prospectively registered with the Australian New Zealand Clinical Trials Registry on 10/11/2022 (ACTRN12622001438741) [14]. Between 2015 and 2019 Diabetes Alliance was implemented in general practices in a staggered and ongoing basis. The pre and post intervention dates differ by practice, creating the stepped wedge design, where every practice switches from control to the intervention (once it had participated in the Diabetes Alliance), just not at the same point in time. Clustering for this study was at a site level, where each site may contain more than one general practice clinic and multiple practice clinicians, with or without Diabetes Alliance training. The data available was at the site level and could not distinguish individual clinics or GPs within each site. De-identified aggregate data were extracted from the NPS MedicineWise MedicineInsight program [15] and restricted for analysis purposes to 13 months prior to the first case conferencing at each site. Analysed data spans April 2014-May 2019 [16]. This study was conducted in accordance with the Declaration of Helsinki [17] and received ethics approval from the Hunter New England Human Research Ethics Committee (15/04/15/5.02). Written, informed consent was obtained from general practices and individually from patients who received the specialist-led case conferencing.

### Recruitment

General practice recruitment for Diabetes Alliance occurred from April 2015 with patient outcomes data included up to May 2019. General practices were recruited via the Hunter New England and Central Coast Primary Health Network website, in their monthly newsletter and via expressions of interest. Recruitment also occurred during the Diabetes Alliance Masterclasses (education sessions) and through word of mouth.

### Participants

For this study, data were extracted for patients 18 years and over with a recorded diagnosis of type 2 diabetes by their GP from practices enrolled in Diabetes Alliance. Data extracted at the practice level included all active patients, defined as 3 or more visits to the practice in the past 2 years, that received care for the management of type 2 diabetes. Participants who received the specialist-led case conferencing (*n* = 1072) were not able to be separated from general practice patients who did not (*n* = 21,634 patients), due to data aggregation. Simulation modelling (described as sensitivity analyses) was used to mimic the removal of the case conference participants from the spillover sample.

### Integrated care intervention

Diabetes Alliance consists of three key activities: (i) case conferencing, (ii) practice feedback, and (iii) education and training.

#### (i) Case conferencing:

Patients enrolled directly in the Diabetes Alliance were provided a face-to-face specialist-led consultation of approximately 45 min duration, at their usual general practice. The case conferencing integrated the diabetes specialist team (endocrinologist, diabetes educator) with the general practice clinicians (GP and practice nurse). During the consultation, complications and comorbidities were reviewed, and a treatment plan was negotiated with the patient. Patients were reviewed by their GP and the diabetes specialist team 6 months after the initial case conference.

#### (ii) Practice feedback:

NPS MedicineWise utilised MedicineInsight data to produce a feedback report for each practice to monitor and evaluate practice-level clinical and process outcomes. The visiting endocrinologist and NPS MedicineWise facilitator discussed the feedback at each practice visit and assisted the practice staff to develop a plan for quality improvement. Practices received feedback reports every 6 months.

#### (iii) Education and training:

Endocrinologists provided 3 × 3 h face-to-face Masterclasses to GPs, registrars, and allied health staff in cycles every 3–4 months per year. Examples of topics included screening, diagnosis, and classification of diabetes, pathophysiology of type 2 diabetes, and medications. Diabetes Educators also provided a full day of education for practice nurses on similar topics.

### Outcomes

Outcomes for this study include compliance with the frequency of diabetes tests. The Annual Cycle of Care recommends that body weight, waist, body mass index (BMI), and blood pressure are monitored every 6 months; HbA1c every 6–12 months; and total cholesterol, low-density lipoprotein (LDL), high-density lipoprotein (HDL), triglycerides, albumin-to-creatinine ratio (ACR) and estimated glomerular filtration rate (eGFR) every 12 months [3]. Clinical outcomes include weight, waist, BMI, blood pressure, HbA1c, total cholesterol, LDL, HDL, triglycerides, ACR, creatinine, and eGFR.

### Statistical analysis

Baseline characteristics were summarised as mean and standard deviation for continuous variables; frequency and percentage for categorical variables. Logistic mixed models were used to test whether the intervention was associated with compliance (yes/no) with test intervals before and after Diabetes Alliance for each outcome, with a random effect for patient within each site to account for the repeated measures of participants and the clustering within sites. The before Diabetes Alliance period began 13 months prior to the start of the intervention at each site, through to 1 month prior (12 months). The after Diabetes Alliance period began 6 months after the last intervention at each site, and lasted 6 months (or 12 months for tests that were only required annually). Linear mixed models were used to test for difference in clinical results (continuous) before and after Diabetes Alliance, with a random effect for patient within site. For outcomes where the residuals violated the assumption of normality, log-transformations were performed, and then results were back-transformed to their original scale. For the mixed models, the mean test value at each stage is reported, along with a p-value for the test of the difference between pre and post mean values.

Estimated glomerular filtration rate could not be adequately modelled in either its original or log transformed form and was therefore categorised and analysed using an ordinal mixed model, with patient within site effects. Odds ratio (OR) and 95% confidence interval (CI) are reported, where the OR represents the odds of scoring a higher eGFR category, where < 15 mL/min/1.73m^2^ is the lowest category, and > 60 mL/min/1.73m^2^ is the highest category (i.e. higher scores indicate better kidney function).

Chi-squared tests were used to assess changes in systolic blood pressure, HbA1c, and LDL cholesterol categories before and after Diabetes Alliance for a subset of patients who had repeated measures, i.e. test values in both the pre and post intervention periods.

Sensitivity analyses were conducted to investigate whether study results reflected spillover effects or if they were driven by the small proportion (< 5%) of patients that received specialist-led case conferencing. Patient-level data was not available, however the number of patients receiving case conferencing at each site was known. Therefore, simulation modelling was used, where a random sample from each site was taken, sampling the number of participants in the spillover: total number for that site minus the number receiving case conferencing at that site. This was performed 200 times until the results converged. The logistic mixed models for HbA1c only (screening tests and clinical result) were then run on each dataset with the mean odds ratio and estimated proportions pre and post reported. Effect estimates were compared to the main analyses. All statistical analyses were performed using Statistical Analysis System (SAS) software (version 9.4; SAS Institute, Cary, North Carolina, United States) and assumed a 5% significance level.

## Results

**Fig. 1:**
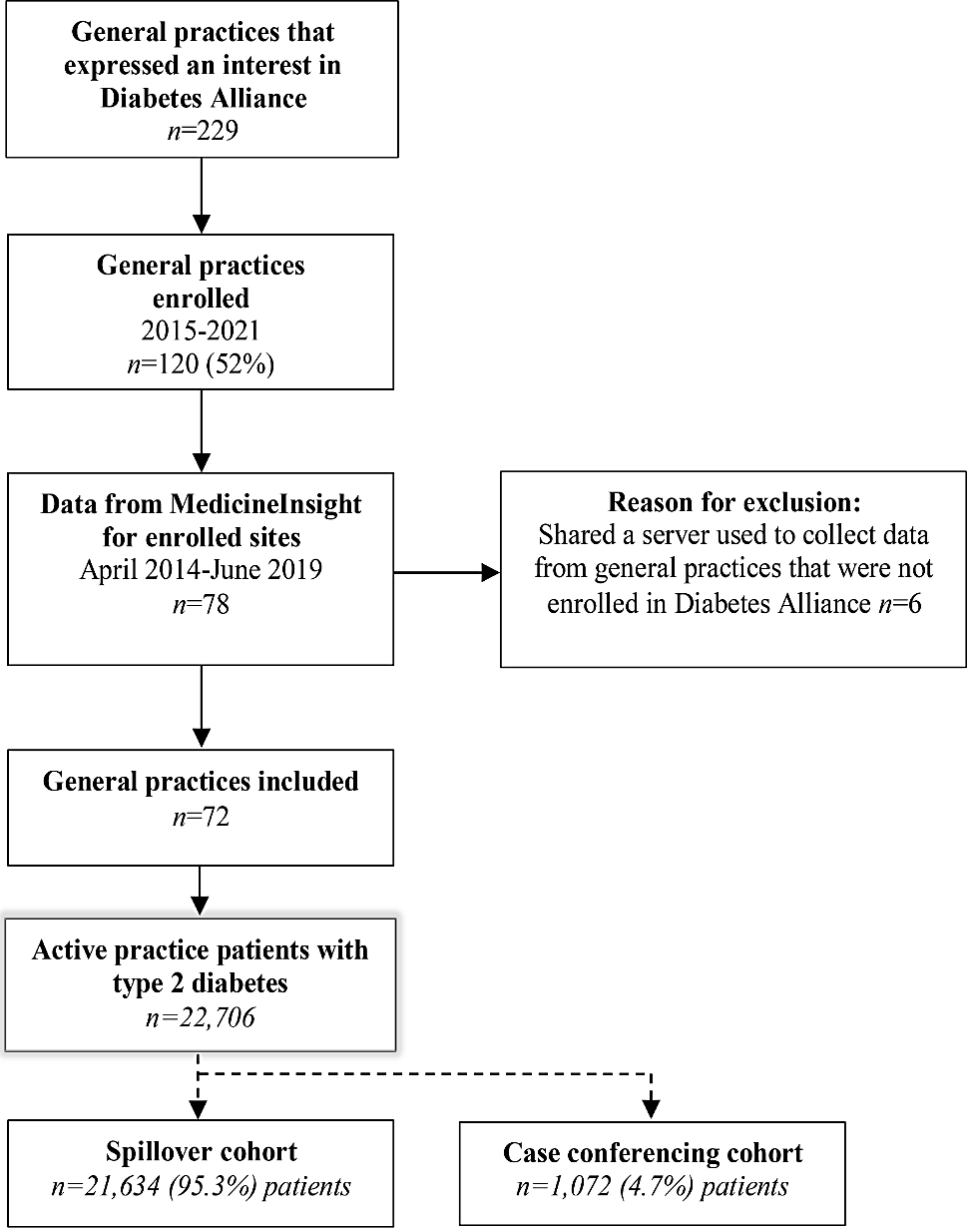
Flow of recruitment of general practices and patients included in the analysis of de-identifiable data

Seventy-two sites, with a total of 22,706 patients with type 2 diabetes were included in the current analyses (Fig. 1). Overall, 1,072 out of 22,706 (4.7%) patients from practices enrolled in Diabetes Alliance directly participated in case conferencing. Characteristics of the patients at included sites are summarised in Table 1. Mean age was 67.7 (±13.5) years and 45% were female. Characteristics of the sample with data at 6 and 12 months post intervention are provided in Supplementary Table 1, Additional File 1 and show negligible differences.

**Table 1:** Characteristics of patients with type 2 diabetes (*n* = 22,706) in Australian general practice sites (*n* = 72) who had received the Diabetes Alliance integrated model of care: snapshot at data transfer, May 2019

Characteristic	Categories	Total (*n* = 22,706)
		Mean (SD)
Age, years		67.70 (13.50)
		**N (%)**
Sex	Male	12,361 (54.44)
	Female	10,323 (45.46)
	Intersex/Indeterminate/Not stated/Not recorded	23 (0.10)
Smoking status	Smoker	2,643 (11.64)
	Ex-Smoker	7,761 (34.18)
	Non-smoker	10,497 (46.23)
	Not recorded	1,805 (7.95)
Remoteness	Inner Regional Australia	7,723 (34.01)
	Major Cities of Australia	12,319 (54.25)
	Outer Regional Australia	2,479 (10.92)
	Remote Australia	89 (0.39)
	Very Remote Australia	10 (0.04)
	Missing	96 (0.42)
Cardiovascular disease	No	16,104 (70.92)
	Yes	6,602 (29.08)
Hypertension	No	7,371 (32.46)
	Yes	15,335 (67.54)

### Frequency of testing

Following Diabetes Alliance, compliance with the recommended testing intervals increased for all 12 clinical outcomes (*p* < 0.0001, Table 2). The largest increases were seen in compliance with measurement of waist circumference (OR 4.45), HbA1c (OR 3.05), BMI (OR 2.45), and weight (OR 2.29).

### Clinical results

Overall, the clinical results showed small but significant improvements after Diabetes Alliance across most of the results, including weight, BMI, systolic and diastolic blood pressure, HbA1c, total cholesterol, LDL cholesterol, and triglycerides (Table 3). There was no difference in waist circumference, and HDL cholesterol reduction was of borderline significance (*p* = 0.05). Urine ACR and creatinine both increased following Diabetes Alliance (Table 3), and there was a reduced odds having an eGFR > 60 after Diabetes Alliance: OR 0.57, 95% CI. 0.52 to 0.63 (p < 0.001). Observed eGFR categories before and after Diabetes Alliance are presented in Supplementary Table 2, Additional File 1.

**Table 2:** Compliance with tests at or above the recommended interval (compliant) before and after Diabetes Alliance: observed frequencies^a^ and logistic mixed models

Screening test	Time interval	Before program	After program	Before program	After program		
	Months	Compliant^a^ *N* (%)	Compliant^a^*N* (%)	Estimated Proportion Compliant (95% CI)	Estimated Proportion Compliant (95% CI)	OR (95% CI)	*P*-value
Weight	6	4,512 (33.96)	6,257 (47.10)	0.27 (0.22, 0.33)	0.46 (0.39, 0.53)	2.29 (2.14, 2.44)	< 0.0001
Height	6	3,481 (26.20)	5,287 (39.80)	-	-	-	-
BMI	6	3,395 (25.56)	5,219 (39.28)	0.18 (0.14, 0.23)	0.35 (0.28, 0.43)	2.45 (2.30, 2.62)	< 0.0001
Waist circum.	6	818 (6.16)	2,445 (18.40)	0.03 (0.02, 0.04)	0.11 (0.08, 0.16)	4.45 (4.04, 4.90)	< 0.0001
BP	6	7,717(58.09)	8,656(65.16)	0.64 (0.58, 0.70)	0.75 (0.70, 0.80)	1.67 (1.56, 1.78)	< 0.0001
HbA1c	6	4,152 (31.25)	6,444 (48.51)	0.24 (0.18, 0.30)	0.48 (0.41, 0.56)	3.05 (2.85, 3.27)	< 0.0001
Total chol.	12	6,564 (53.26)	7,714 (62.59)	0.53 (0.44, 0.62)	0.67 (0.59, 0.75)	1.81 (1.69, 1.93)	< 0.0001
LDL	12	5,772 (46.84)	6,559 (53.22)	0.41 (0.33, 0.51)	0.50 (0.40, 0.59)	1.41 (1.32, 1.49)	< 0.0001
HDL	12	5,568 (45.18)	6,298 (51.10)	0.44 (0.35, 0.53)	0.53(0.44, 0.62)	1.44 (1.36, 1.53)	< 0.0001
Triglycerides	12	6,543 (53.09)	7,689 (62.39)	0.53 (0.44, 0.62)	0.67 (0.58, 0.75)	1.80 (1.69, 1.92)	< 0.0001
ACR	12	4,508 (36.58)	5,689 (46.16)	0.31 (0.24, 0.39)	0.45 (0.36, 0.54)	1.81 (1.70, 1.93)	< 0.0001
eGFR	12	7,101 (57.62)	8,477 (68.78)	0.61 (0.52, 0.70)	0.78 (0.70, 0.84)	2.19 (2.04, 2.35)	< 0.0001
Creatinine	12	7,177 (58.24)	8,541 (69.30)	0.62 (0.53, 0.71)	0.78 (0.71, 0.84)	2.18 (2.04, 2.34)	< 0.0001

BMI, Body Mass Index, BP, blood pressure; CI, confidence interval; eGFR, estimated glomerular filtration rate; N, number; OR, odds ratio; total chol, total cholesterol; waist circum, waist circumference

^a^ Includes sites with a full 12 months pre data, and post period defined to be 6 months for outcomes where the minimum test interval is 6-monthly (40 sites), and 12 months where minimum test interval is 12-monthly (36 sites)

For log-transformed outcomes, absolute differences were also calculated (Table 3), but these values only hold at the mean, and the results are multiplicative, meaning at higher values the differences are greater and at lower values these differences are smaller. For a HbA1c of 10%, we expect the change to be 10 × 0.996 = 9.96%, i.e. a reduction in HbA1c of 0.04% after the Diabetes Alliance intervention. With a mean weight of 90.19 kg before Diabetes Alliance and 88.81 kg after, there was an absolute improvement at the mean of -1.38 kg. With a mean of 6.84% for HbA1c before the Diabetes Alliance and 6.81% after the intervention, there was an absolute improvement of -0.03% for HbA1c at the mean, and − 0.04% at a HbA1c of 10% (10 × 0.996 = 9.96; a change of -0.04%).

**Table 3:** Clinical outcomes before and after Diabetes Alliance integrated care in general practices

		Before program	After program		
Clinical outcome	*N*	Mean estimate (95% CI)^1^	Mean estimate (95% CI)^1^	Difference in means (95% CI)^2^	*P*-value
Weight (kg)	14,882	90.19 (89.85, 90.53)	88.81 (88.47, 89.16)	-1.38 (-1.38, -1.87)	< 0.0001
Waist circum. (cm)	7,113	109.80 (109.40, 110.20)	109.50 (109.10, 109.90)	-0.28 (-0.59, 0.03)	0.0764
BMI (kg/m^2^)	13,111	32.24 (32.13, 32.35)	31.80 (31.69, 31.92)	-0.44 (-0.44, -0.44)	< 0.0001
Systolic BP (mmHg)	17,963	135.80 (135.60, 136.00)	134.70 (134.50, 135.00)	-1.12 (-1.33, -0.91)	< 0.0001
Diastolic BP (mmHg)	17,966	77.07 (76.94, 77.21)	75.90 (75.74, 76.06)	-1.18 (-1.30, -1.05)	< 0.0001
HbA1c (%)	15,144	6.84 (6.82, 6.86)	6.81 (6.79, 6.83)	-0.03 (-0.03, -0.03)	0.0021
HbA1c (mmol/mol)	15,144	50.70 (50.50, 50.90)	50.43 (50.20, 50.66)	-0.27 (-0.27, -0.27)	0.0041
ACR (mg/mmol)	11,081	1.77 (1.72, 1.82)	1.84 (1.78, 1.90)	0.07 (0.07, 0.07)	0.0072
Total chol. (mmol/L)	14,878	4.32 (4.30, 4.34)	4.22 (4.20, 4.24)	-0.11 (-0.10, -0.10)	< 0.0001
LDL (mmol/L)	12,432	2.17 (2.16, 2.19)	2.07 (2.05, 2.09)	-0.10 (-0.10, -0.10)	< 0.0001
HDL (mmol/L)	12,926	1.15 (1.15, 1.16)	1.15 (1.14, 1.16)	-0.01 (-0.01, -0.01)	0.0494
Triglycerides (mmol/L)	14,803	1.77 (1.76,1.79)	1.76 (1.74,1.77)	-0.02 (-0.02, -0.02)	0.0078
Creatinine (µmol/L)	16,072	82.60 (82.20, 83.00)	84.67 (84.22, 85.11)	2.07 (2.07, 2.08)	< 0.0001

BP, blood pressure; chol, cholesterol; circum, circumference; cm, centimetre; kg, kilogram; L, litre; m, metre; mmHg, millimetre of mercury; mmol, millimole; µmol, micromole

^1^Weight, BMI, HbA1c (percentage and mmol/.mol), ACR, cholesterol, LDL, HDL, Triglycerides, and Creatinine were all log-transformed to meet normality assumptions

^2^Weight, BMI, HbA1c, ACR, cholesterol, LDL, HDL, Triglycerides, and Creatinine were back-transformed. Estimated difference only valid at the means (at higher reference values of the outcome the estimated difference will be greater, and at smaller reference values the difference will be smaller)

### Changes in clinical results categories for subset with repeated measures

**Fig. 2:**
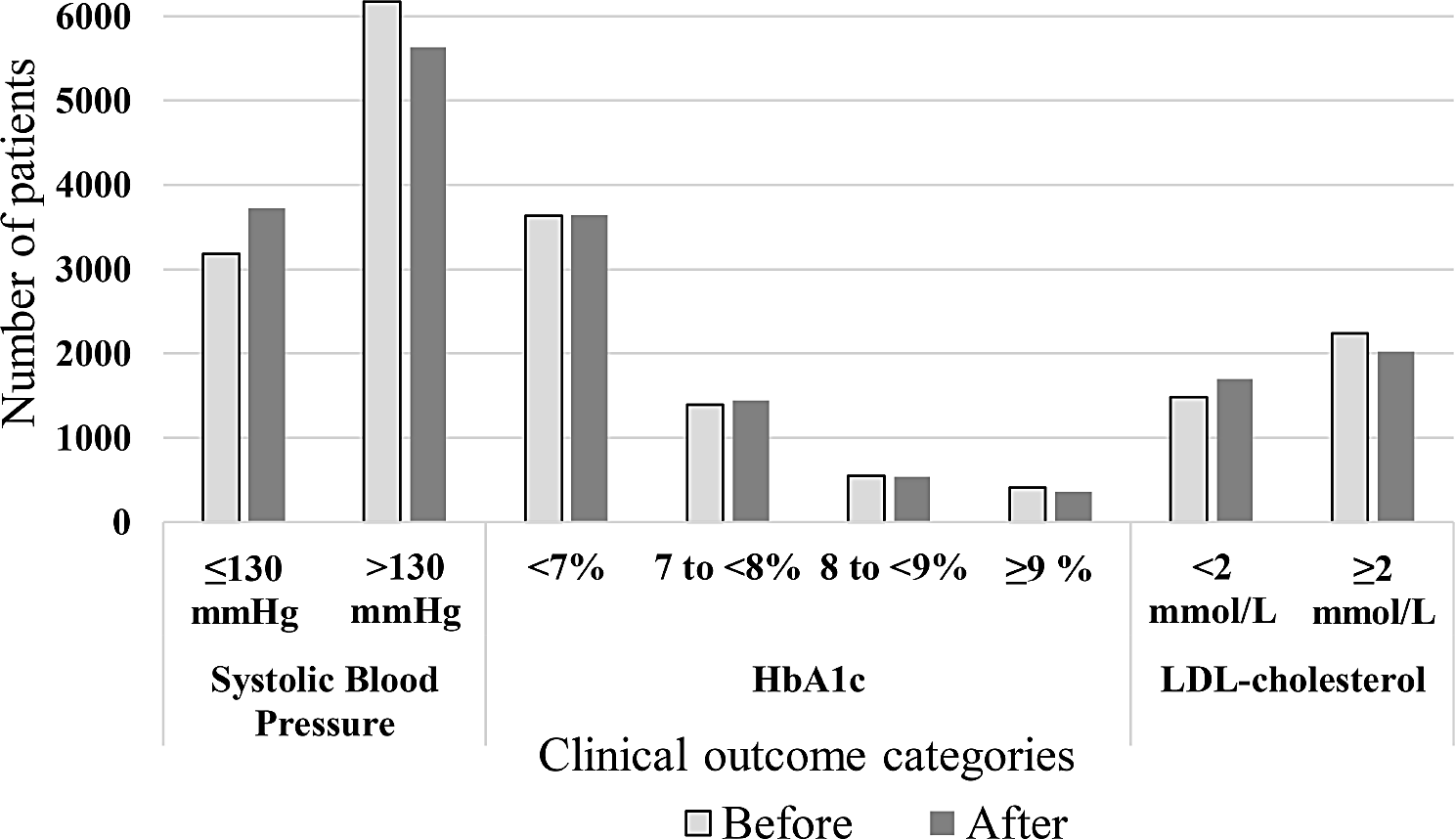
Change in clinical outcome categories for systolic blood pressure, HbA1c and LDL-cholesterol before (light grey) and after (dark grey) Diabetes Alliance for patients with complete data

For the subset of patients with test results both before and after the Diabetes Alliance intervention, significant changes in clinical categories were observed for blood pressure, HbA1c, and LDL-cholesterol (Fig. 2, Supplementary Table 3, Additional File 1). Whilst most patients did not change category, a higher proportion of patients moved down to the lowest category (i.e. met the clinical target) in systolic blood pressure, HbA1c, and LDL-cholesterol, than moved up to the highest. For blood pressure (*n* = 9360, 41% of total sample), 18% moved down to ≤ 130 mm Hg systolic blood pressure after Diabetes Alliance, whereas only 12% moved up to > 130 mm Hg systolic blood pressure. For HbA1c (*n* = 5989, 26% of total sample), 18% moved down to a lower HbA1c category (< 7%, 7 to ≤ 8%, or 8 to ≤ 9%) after the intervention while 17% moved up (7 to ≤ 8%, 8 to ≤ 9%, or > 9%). For LDL-cholesterol (*n* = 3715, 16% of total sample), 13% of patients moved down to < 2mmol/L after Diabetes Alliance, and only 7% moved up to ≥ 2mmol/L.

### Sensitivity analysis

Results from the simulation models indicated very slightly attenuated effect estimates for HbA1c. Patients were 2.29 (2.31, 2.65) times more likely to have their HbA1c levels measured at 6 months after Diabetes Alliance. This is lower than the estimate from the main analysis [3.05 (2.85–3.27)], but still significantly greater than 1. The proportion of patients that received 6 monthly HbA1c tests was 0.27 (0.26, 0.28) before the program and 0.48 (0.47, 0.49) after Diabetes Alliance. These proportions are also close to the point estimates of 0.24 and 0.48 from the main analysis. The mean test value in the pre period of 6.83% (95% CI 6.82–6.84%) and 6.81% (95% CI 6.80–6.82%) in the post period are also very similar to the values from the main analysis (6.84% and 6.81%, respectively), suggesting the presence of a spillover effect is robust.

## Discussion

Overall, the Diabetes Alliance integrated model of care was associated with significant improvements in the frequency of diabetes screening tests, and small but significant improvement in clinical outcomes including weight, BMI, blood pressure, blood lipid profiles and glycaemic control across up to 22,706 general practice patients with type 2 diabetes. The learnings from the specialist-led case conferencing delivered to a small proportion of patients, accompanied by other capability building activities, spilled over to the wider cohort of general patients receiving usual care without direct involvement from the specialist team. These results are in keeping with previous small pilot studies [18, 19] and prospective controlled trials [20–22], and have now been confirmed at scale across a broad geographic footprint.

Increased testing does not automatically translate to better healthcare; however, statistically significant improvements in most of the clinical results were observed for all patients with type 2 diabetes across the general practice sites who had received the Diabetes Alliance intervention. If sustained, these improvements should translate into reduced morbidity and mortality at the population level. Results indicated that Diabetes Alliance was associated with a reduction in HbA1c even at the mean value of 6.84%, which is within recommendations, noting that slightly higher reductions occurred at higher HbA1C values. While the spillover benefits were modest when each clinical outcome was considered separately, the combination of clinical benefits (i.e. improved weight, blood pressure, lipids and glycaemic control) is likely to be synergistic and significant at a population level. A simulation study demonstrated that reductions in HbA1c levels by as little as 0.1%, up to 0.8% (in 0.1% increments), in a Swedish population with type 1 diabetes reduced the incidence of microvascular complications, improved life expectancy and quality of life over a 50-year time horizon, illustrating the importance of early improvement in glycaemic control, even if modest [23]. While glucose control remains a major focus in the management of patients with type 2 diabetes [24], the synergistic effects of improved blood pressure, blood lipids, and HbA1c likely confers the greatest benefit for reducing cardiovascular disease risk [25]. Diabetes Alliance is a real-world intervention that takes into consideration the “prevention paradox”, where a large number of individuals with less risk creates more cases in absolute terms than a small number of individuals with higher risk [26] The spillover effect show here is on all patients, the majority of whom had no interaction with the Diabetes Alliance program directly. The integrated care model specifically aims to enhance general practitioners capability in managing diabetes so the emphasis is on small but sustained improvements in the majority of patients, rather than the minority who are seen during specialist-led case conferencing or in hospital. The aim is to shift the distribution curve for a large sample, toward a favourable outcome, which has been demonstrated by the findings of this study.

The clinical results for ACR, creatinine, and eGFR observed in this study are consistent with kidney function declining over time for patients with type 2 diabetes; slowing the rate of decline represents improvement. The rate of kidney function decline has been studied recently in a cohort of 32,492 adult patients with type 2 diabetes (mean age 66.3 years, 52.6% male) predominantly located in Germany [27]. Over three years, 31% of patients had an eGFR slope of -12 mL/min/1.73m^2^ or more, and the proportion of patients with an eGFR < 30 mL/min/1.73m^2^ more than doubled [27]. Rates of chronic kidney disease in the Hunter New England region are high by Australian standards, identified as one of the top 20 hot spots contributing to the national burden of kidney disease [28]. The increase in urine ACR and creatinine in our study likely reflects the increased screening following Diabetes Alliance, noting creatinine/eGFR had more than twice the odds of being tested after Diabetes Alliance as it did before the intervention. Some reduction in eGFR may also be the result of an increased initiation of angiotensin-converting enzyme (ACE) inhibitors, angiotensin II receptor blockers (ARBs) and sodium-glucose co-transporter-2 (SGLT2) inhibitors, where kidney function shows an initial decline with medication commencement but nephroprotective effects over time [29].

This study has evaluated the real-world spillover effect of an integrated model of care that has been implemented at scale across one Australian local health district. The nonrandomised pragmatic trial included a large sample of patients with type 2 diabetes from general practices using data from MedicineInsight, a national database. The analyses on the subset of patients with repeated measures looking at changes in clinical categories is at risk of selection bias and results need replication in other studies to be generalisable. We were also not able to identify the 4.7% of patients who had directly received case conferencing and exclude them to define a clean spillover sample. Instead, we ran simulation models for HbA1c to mimic patient removal and findings remained significant and consistent with the main analyses. Another limitation is the inability to quantify the extent of Diabetes Alliance intervention received by each site. General Practices vary considerably in size, both in number of GPs and patients with type 2 diabetes. Inclusion of sites with a mix of GPs who had and had not engaged with one or more Diabetes Alliance intervention activities, would likely bias our results towards the null, meaning the true effect estimates would be greater if restricted to GPs (and their spillover patient group) who had actively participated. Future evaluation of the Diabetes Alliance also aims to include a parallel control group; however, this was not feasible at the time of these analyses.

## Conclusions

Although Diabetes Alliance was associated with significant improvements in the frequency of screening tests and clinical results across a large cohort, it is unclear if these effects are maintained over the longer-term and what impact they may have on reducing micro- and macrovascular complications. Hence, further research and evaluation is needed to determine the health, societal, and economic impacts from this real-world, integrated model of care for diabetes.

## Electronic supplementary material

Below is the link to the electronic supplementary material.

Supplementary Material 1

## Data Availability

The author confirms that all data generated or analysed during this study are included in this published article and Additional File 1.

## Abbreviations

ACR: Albumin-to-Creatinine Ratio
ACE: Angiotensin-Converting Enzyme
ARBs: Angiotensin II Receptor Blockers
BMI: Body Mass Index
CI: Confidence Interval
eGFR: Estimated Glomerular Filtration Rate
GP: General Practitioners
HDL: High-Density Lipoprotein
HNELHD: Hunter New England Local Health District
LDL: Low-Density Lipoprotein (LDL)
OR: Odds Ratio
SAS: Statistical Analysis System
SGLT2: Sodium-Glucose Co-Transporter-2
