# Supplementary material for: Spillover effects from a type 2 diabetes integrated model of care in 22,706 Australians: an open cohort stepped wedge trial
Source: BMC Endocr Disord. 2024 Sep 10;24:183. doi: 10.1186/s12902-024-01692-4 (PMC11384678; doi:10.1186/s12902-024-01692-4)
Supplement: Supplementary file 1 — Supplementary Material 1 [file 12902_2024_1692_MOESM1_ESM.docx]

**Additional files**

**File name:** Additional File 1

**File format:** Microsoft Word (DOC)

**Title of data:**

**Supplementary Table 1:** Characteristics of patients with type 2 diabetes in Australian general practice sites who had received the Diabetes Alliance integrated model of care: snapshot at data transfer (May 2019), restricted to those with at least 6 and 12 months follow-up

**Supplementary Table 2:** Observed Estimated Glomerular Filtration Rate (eGFR) categories before and after the Diabetes Alliance

**Supplementary Table 3:** Change in clinical outcome categories after Diabetes Alliance for patients with complete data

**Description of data:**

Contains three supplementary tables that describes characteristics of patients, observed Estimated Glomerular Filtration Rate (eGFR) categories, clinical outcome categories of patients with complete data after the intervention

**Additional File 1**

**Supplementary Table 1:** Characteristics of patients with type 2 diabetes in Australian general practice sites who had received the Diabetes Alliance integrated model of care: snapshot at data transfer (May 2019), restricted to those with at least 6 and 12 months follow-up

| **Variable** | **Categories** | **6 months,  N=13,285** | **12 months N=12,324** |
| --- | --- | --- | --- |
|  |  | **Mean (SD)** |  |
| Age, years |  | 67.9 (13.6) | 67.8 (13.7) |
|  |  | **n (%)** |  |
| Sex | Male | 7,291 (55.0) | 6,717 (55.0) |
|  | Female | 5,983 (45.0) | 5,597 (45.0) |
|  | Intersex/Indeterminate/Not stated/Not recorded | 11 (0.1) | 10 (0.1) |
| Patient Status | Active | 12,195 (92.0) | 11,243 (91.0) |
|  | Deceased | 345 (2.6) | 342 (2.8) |
|  | Inactive | 741 (5.6) | 735 (6.0) |
|  | Visitor | 4 (0.0) | 4 (0.0) |
| Smoking | Smoker | 1,549 (12.0) | 1,458 (12.0) |
|  | Ex-Smoker | 4,603 (35.0) | 4,230 (34.0) |
|  | Non-smoker | 6,193 (47.0) | 5,769 (47.0) |
|  | Not recorded | 940 (7.1) | 867 (7.0) |
|  | Missing | 0 (0.0) | 0 (0.0) |
| Remoteness | Inner Regional Australia | 4,178 (32.0) | 3,785 (31.0) |
|  | Major Cities of Australia | 7,461 (56.0) | 6,935 (57.0) |
|  | Outer Regional Australia | 1,496 (11.0) | 1,462 (12.0) |
|  | Remote Australia | 78 (0.6) | 77 (0.6) |
|  | Very Remote Australia | 9 (0.1) | 8 (0.1) |
|  | Missing | 63 (0.5) | 57 (0.5) |
| Cardiovascular disease | No | 9,296 (70.0) | 8,643 (70.0) |
|  | Yes | 3,989 (30.0) | 3,681 (30.0) |
|  | Missing | 0 (0.0) | 0 (0.0) |
| Hypertension | No | 4,282 (32.0) | 3,985 (32.0) |
|  | Yes | 9,003 (68.0) | 8,339 (68.0) |
|  | Missing | 0 (0.0) | 0 (0.0) |

**Supplementary Table 2:** Observed Estimated Glomerular Filtration Rate (eGFR) categories before and after the Diabetes Alliance

| **eGRF category**  **(mL/min/1.73m^2^)** | **Before**  **n (%)** | **After**  **n (%)** | **Total**  **N** |
| --- | --- | --- | --- |
| <15 | 135 (1.0%) | 124 (1.3%) | 259 |
| 15-<30 | 754 (4.5%) | 527 (5.6%) | 1281 |
| 30-60 | 4223 (25.3%) | 2572 (27.1%) | 6795 |
| >60 | 11558 (69.3%) | 6253 (65.9%) | 17811 |

**Supplementary Table 3.** Change in clinical outcome categories after Diabetes Alliance for patients with complete data

| **Before Diabetes Alliance** | | **After Diabetes Alliance, n (row %)** | | | | **P-value** |
| --- | --- | --- | --- | --- | --- | --- |
| **SBP category** | N=9,360 | **SBP ≤130 mmHg** | **SBP >130 mmHg** |  |  |  |
| ≤130 mmHg | 3183 | 2,064 (64.8) | 1,119 (35.2) |  |  |  |
| > 130 mmHg | 6177 | 1,662 (26.9) | 4,515 (73.1) |  |  | <0.0001 |
| **HbA1c category** | N=5,989 | **<7%** | **7 to ≤8%** | **8 to ≤9%** | **≥9%** |  |
| <7% | 3634 | 2,959 (81.4) | 535 (14.7) | 91 (2.5) | 49 (1.4) |  |
| 7 to ≤8% | 1393 | 508 (36.5) | 612 (43.9) | 198 (14.2) | 75 (5.4) |  |
| 8 to ≤9% | 551 | 113 (20.5) | 203 (36.8) | 153 (27.8) | 82 (14.9) |  |
| ≥9% | 411 | 63 (15.3) | 96 (23.4) | 93 (22.6) | 159 (38.7) | <0.0001 |
| **LDL category** | N=3,715 | **LDL <2** **mmol/L** | **LDL ≥2** **mmol/L** |  |  |  |
| <2 mmol/L | 1478 | 1,207 (81.7) | 271 (18.3) |  |  |  |
| ≥2 mmol/L | 2237 | 490 (21.9) | 1,747 (78.1) |  |  | <0.0001 |

HbA1c, Glycosylated haemoglobin; LDL, Low-density lipoprotein; SBP, Systolic blood pressure
